# Ventilatory Chaos Is Impaired in Carotid Atherosclerosis

**DOI:** 10.1371/journal.pone.0016297

**Published:** 2011-01-28

**Authors:** Laurence Mangin, Guy Lesèche, Alain Duprey, Christine Clerici

**Affiliations:** 1 Assistance Publique-Hôpitaux de Paris, Hôpital Bichat-Claude Bernard, Service de Physiologie, Paris, France; 2 Université Denis Diderot-Paris 7, Paris, France; 3 Laboratoire Matières et Systèmes Complexes (MSC), UMR 7057, CNRS, Paris, France; 4 Assistance Publique-Hôpitaux de Paris, Hôpital Bichat-Claude Bernard, Service de Chirurgie Thoracique, Vasculaire et Transplantation Pulmonaire, Paris, France; 5 Inserm, U773, CRB3, Paris, France; Queensland Institute of Medical Research, Australia

## Abstract

Ventilatory chaos is strongly linked to the activity of central pattern generators, alone or influenced by respiratory or cardiovascular afferents. We hypothesized that carotid atherosclerosis should alter ventilatory chaos through baroreflex and autonomic nervous system dysfunctions. Chaotic dynamics of inspiratory flow was prospectively evaluated in 75 subjects undergoing carotid ultrasonography: 27 with severe carotid stenosis (>70%), 23 with moderate stenosis (<70%), and 25 controls. Chaos was characterized by the noise titration method, the correlation dimension and the largest Lyapunov exponent. Baroreflex sensitivity was estimated in the frequency domain. In the control group, 92% of the time series exhibit nonlinear deterministic chaos with positive noise limit, whereas only 68% had a positive noise limit value in the stenoses groups. Ventilatory chaos was impaired in the groups with carotid stenoses, with significant parallel decrease in the noise limit value, correlation dimension and largest Lyapunov exponent, as compared to controls. In multiple regression models, the percentage of carotid stenosis was the best in predicting the correlation dimension (p<0.001, adjusted R^2^: 0.35) and largest Lyapunov exponent (p<0.001, adjusted R^2^: 0.6). Baroreflex sensitivity also predicted the correlation dimension values (p = 0.05), and the LLE (p = 0.08). Plaque removal after carotid surgery reversed the loss of ventilatory complexity. To conclude, ventilatory chaos is impaired in carotid atherosclerosis. These findings depend on the severity of the stenosis, its localization, plaque surface and morphology features, and is independently associated with baroreflex sensitivity reduction. These findings should help to understand the determinants of ventilatory complexity and breathing control in pathological conditions.

## Introduction

Human ventilatory rhythmogenesis arises from the brainstem and the medulla [1]. It depends on phasic neuronal activities taking place within central respiratory generators located in the brain stem and on their transformation into rib cage movements by the respiratory muscles. Nonlinear dynamics in the ventilatory flow output arises as a result of the complex interplay between central processing of the respiratory centers, peripheral afferents [2] and stochastic noise inputs. When adequately stimulated, neural population from the pre-Bötzinger complex exhibits in vitro an oscillatory neural activity like periodicity, mixed-mode oscillations, quasiperiodicity and ultimately disorganized aperiodic activity [3], a typical transition to chaos. However, peripheral afferents have been shown to play a role in the nonlinear dynamics of ventilation. Sammon et al [4], [5] showed that vagal afferent activity increases ventilatory complexity. In mechanically ventilated rats, irregular inspiratory-expiratory phase switching and central respiratory pattern generator output are consistent with low-dimensional chaos, probably correlated with lung deflation [5]. In unsedated humans receiving mechanical ventilation, ventilatory flow has been shown to exhibit chaotic properties arising from the intrinsic properties of the respiratory central pattern generator in response to vagal afferent feedbacks [2], [6]. Taken together these findings point out that ventilatory chaos is strongly linked to the activity of central pattern generators, alone or influenced by respiratory or cardiovascular afferents [6], via autonomic nervous system. It thus provides a sensitive means of assessing breathing control.

Carotid atherosclerosis has a high prevalence in adults with cardiovascular risk factors [7]. Such carotid lesions have been shown to perturb baroreflex sensitivity as well as autonomic nervous system activity [8]–[10]. Carotid atheroma induces locally structural changes that decrease the arterial distensibility [11] with impairment of baroreflex sensitivity [9], [10]. The presence of carotid atheroma may also impair ventilatory flow characteristics through several pathways. The carotid plaque targets the carotid sinus, a structure known to be involved in the regulation of respiration [12]. The baroreflex controls ventilation through changes in the frequency [12], tidal volume [12], [13] and breathing variability [14]. Moreover, autonomic nervous system dysfunction occurs in the presence of a greater intima-media thickness of the carotid arteries [9] and during carotid atherosclerosis [10]. This sympatho-vagal imbalance could contribute to breathing control abnormalities as well. Surprisingly, until now no study has been done to evaluate the consequences of carotid atherosclerosis on ventilatory flow dynamics.

The aims of the study were: (i) to characterize linear and nonlinear features of inspiratory flow dynamics according to the severity of the carotid stenosis, its localization and characteristics of the plaque, (ii) to determine whether these changes correlate with baroreflex sensitivity, (iii) to study the effects of plaque removal on inspiratory flow chaos abnormalities.

## Results

### Subject characteristics

We recruited 75 patients. Twenty five were in the control group, 23 in the group with moderate carotid stenosis, and 27 in the group with severe carotid stenosis. The clinical characteristics of the patients are summarized in Table 1. There were 50 men and 25 women. Age was significantly higher in the group with severe stenosis as compared to control subjects (p<0.05). No significant differences were noted between the 3 groups concerning clinical data, i.e. body mass index, smoking status, diabetes and hypertension. As expected, the degree of carotid stenosis was significantly higher in the groups with moderate (p<0.001) and severe (p<0.001) stenoses compared to control subjects. Six subjects had a history of transient ischemic attack, all in the group with severe stenosis. Lung function and oxygen saturation were normal in all three groups. The carotid plaque was heterogeneous in 64% of cases and irregular in 48% of cases. Its localization was unilateral in 40% of cases, concerned the internal carotid alone in 56% of cases. Baroreflex sensitivity was significantly lower in the groups with moderate and severe stenosis, as compared with controls (moderate stenosis: 3±1.8ms/mmHg and severe stenosis: 3±1.7ms/mmHg *versus* Controls: 4.6±2.5 ms/mmHg, p = 0.01).

**Table 1:** Clinical characteristics and lung function of the subjects.

	Control group	Moderate stenosis	Severe stenosis
	*(n = 25)*	*(n = 23)*	*(n = 27)*
**Clinical characteristics**			
Age (yr)	62±12	68±8	69±9*
Gender (M/F)	13/12	17/6	20/7
Body mass index (kg/m^2^)	26±4	26±3	25±3
Current Smokers	5	3	6
Diabetes	5	9	6
Hypertension	14	13	21
Transient ischemic attack	0	0‡‡‡	6***
Carotid stenosis (%)	0	39±12‡‡‡	72±22***
**Lung function**			
FEV1 (%predicted)	96±14	90±18	85±13
FEV1/FVC (%predicted)	100±17	101±6	104±10
FEF 75 (%predicted)	106±23	102±30	92±24
Oxygen Saturation (%)	98±1	98±2	97±2

Values are mean±SD; FEV1: forced expiratory volume in the first second, FEV1/FVC: forced expiratory volume in the first second/forced vital capacity, FEF75: 75% of the forced expiratory flow;

*p<0.5,

***p<0.001, Control group versus Severe stenosis;

‡‡‡ p<0.001, Moderate stenosis versus Severe stenosis; Anova1 or χ^2^ tests when appropriate.

### Linear measures of the ventilatory variables

The means of the Ti, Te, Ttot, Vt, Ti/Ttot, Vt/Ti were not significantly different between the three groups. Subjects with carotid stenosis breathed with a significantly higher coefficient of variation of the Ti, Te, Ttot, Vt, Ti/Ttot, Vt/Ti (Table 2). No significant alteration of the breath variations was evidenced in the group with severe stenosis between subjects with a history of transient ischemic attack and the rest of the group. Autocorrelation coefficient of the inspiratory flow significantly increased in the group with moderate stenosis, as compared with controls (Table 2).

**Table 2:** Linear measures, coefficients of variation and autocorrelation coefficient, of breath variables among the three groups.

	Control group	Moderate Stenosis	Severe Stenosis
	*(n = 25)*	*(n = 23)*	*(n = 27)*
***Coefficient of variation***			
Inspiratory time (s)	0.11±0.04	0.15±0.06†††	0.17±0.06***
Expiratory time	0.13±0.03	0.15±0.05‡‡‡	0.18±0.05***
Total Cycle time	0.10±0.04	0.13±0.04†††	0.15±0.04***
Tidal Volume (l)	0.14±0.07	0.16±0.07	0.21±0.10**
Total duty cycle	0.08±0.03	0.10±0.04	0.11±0.03**
Inspiratory flow (l/s)	0.13±0.04	0.14±0.06	0.16±0.11*
***Autocorrelation coefficient one breath lag***			
Inspiratory flow	0.7±0.22	0.83±0.12†	0.77±0.21

Values are mean±SD;

***p<0.001,

**p<0.01,

*p<0.05 Control group versus Severe stenosis;

† p<0.05,

††† p<0.001 Control versus Moderate stenosis,

‡‡‡ p<0.001, Moderate versus Severe stenosis. Covariance analysis controlling for age.

Unilateral or bilateral stenosis significantly increased the CV of the Ttot (p<0.001), Ti (p<0.001), Te (p<0.01), Vt (p = 0.05), Ti/Ttot (p = 0.01) (Figure S1). Localization of the stenosis to the bifurcation or the internal carotid significantly increased the CV of: Ti (p<0.001), Ttot (p<0.001), Te (p<0.01), Vt (p = 0.001), Ti/Ttot (p = 0.01), Vt/Ti (p = 0.01) (Figure S2). Characteristics of the carotid plaque such as irregularity or heterogeneity significantly modified the breath variables in the time domain (Figure S3). Autocorrelation coefficient at one breath lag of the inspiratory flow was higher in case of a heterogeneous carotid stenosis (p<0.01).

N way-analyses of variance considering all the characteristics of the plaque (percentage of carotid stenosis, heterogeneity, irregularity, bilaterality, involvement of the bifurcation) revealed that the percentage of the carotid stenosis had the main significant effect on the CV of Vt (p<0.001) and Vt/Ti (p<0.001). As shown in table 3, significant correlations were found between the degree of carotid stenosis and breathing variability parameters in the time domain (Ti, Te, Ttot, Vt/Ti). Among the other characteristics, the plaque heterogeneity (compared to homogeneity) and a carotid stenosis involving the carotid bifurcation (compared to internal carotid stenosis) had significant effects on the CV of Vt, Vt/Ti (p<0.001), Ti/Ttot (p = 0.05) and on the autocorrelation coefficient of the inspiratory flow (p<0.001).

**Table 3:** Correlation coefficients between carotid stenosis and linear/nonlinear measures of breath variations and baroreflex sensitivity.

	Carotid stenoses	p value
	(%)	
***Linear analyses of breath components***		
*Coefficients of variations*		
Inspiratory time	0.4	p<0.001
Expiratory time	0.4	p<0.001
Total cycle time	0.4	p<0.001
Inspiratory flow	0.3	p = 0.02
*Autocorrelation coefficient one breath lag*		
Inspiratory flow	0.2	p = 0.07
***Non linear analyses of inspiratory flow***		
Noise limit	−0.35	p<0.01
Correlation Dimension	−0.6	p<0.001
Largest Lyapunov exponent	−0.7	p<0.001
***Baroreflex sensitivity***	−0.3	p = 0.02

Partial correlation coefficient are given controlling for age.

Multiple regression analyses including the degree of carotid stenosis, the age and the baroreflex sensitivity as independent variables and the successive breath parameters, as dependent variables revealed that carotid stenosis had the highest predictive value for predicting the variability of Ttot (p = 0.01, adjusted R^2^:0.15), Ti (p<0.01, adjusted R^2^:0.2), Te (p<0.01, adjusted R^2^: 0.2).

### Nonlinear measures of the ventilatory variables

**Figure 1:**
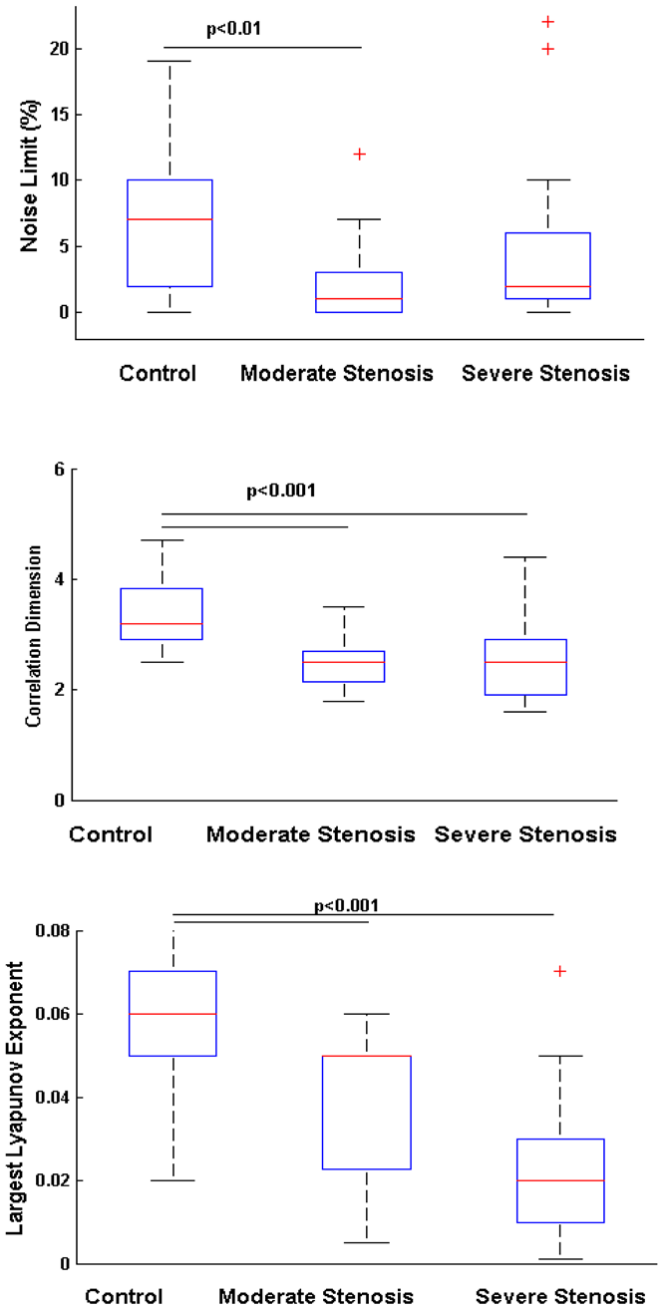
Chaos characterization of inspiratory flow in the control group, in the groups with moderate and severe carotid stenosis. Noise Limit value (%) in the top, Correlation dimension in the middle and largest Lyapunov exponent in the bottom. The boxes encompass the interquartile range with indication of the median, the whiskers delimit the 95^th^ percentile of the data distribution (ANOVA).

Positive noise limit values characterizing chaos were found in 23/25 time series of inspiratory flow in the control group, in 12/23 time series in the group with moderate stenosis and 22/27 in the group with severe stenosis (p<0.01, χ^2^ test). Noise limit values were lower in the group with moderate stenosis, as compared with controls (p<0.01) (Figure 1). We therefore evaluated the correlation dimension and largest Lyapunov exponent (LLE) of the inspiratory flow in the 57 time series that exhibit a positive noise limit value. The correlation dimension and the LLE were lower in the groups with moderate and severe stenoses (Figure 1). Bilateral stenosis significantly decreased the noise limit value as compared with no stenosis (p<0.05). Either bilateral or unilateral stenoses significantly reduced the correlation dimension and LLE of the inspiratory flow (Figure S4). Bifurcation or internal carotid stenoses also decreased the correlation dimension and LLE compared with controls (p<0.001) (Figure S4). Irregularity or heterogeneity also modified the chaotic features of the inspiratory flow (Figure S4). N way-analyses of variance considering all the characteristics of the plaque revealed that the stenosis severity (percentage of stenosis) had the main significant effect on the LLE (p = 0.03). As shown in table 3 in univariate analysis, significant correlations were found between the degree of carotid stenosis and the nonlinear analyses of the ventilatory flow (the noise limit value, correlation dimension and LLE).

Multiple regression analyses including in the model, the degree of carotid stenosis, the age and the baroreflex sensitivity, as independent variables and the correlation dimension and LLE, as successive dependent variables showed that the carotid stenosis was the best in predicting the chaotic features of the inspiratory flow (correlation dimension: p<0.001, adjusted R^2^: 0.35; LLE: p<0.001, adjusted R^2^: 0.6). Also in the multiple regression model, baroreflex sensitivity had a significant effect in predicting the correlation dimension values (p = 0.05), and to a lesser extent the LLE (p = 0.08).

The correlation dimensions of the 57 experimental time series were compared with 5 surrogates (290 simulated time series) that match each original signal. Those surrogates were computed after assigning random phase. Significant differences were obtained between the original data paired with the corresponding average correlation dimension values form the matching surrogate (p<0.001, Wilcoxon signed-rank test), reinforcing the nonlinear feature of the inspiratory flow.

**Figure 2:**
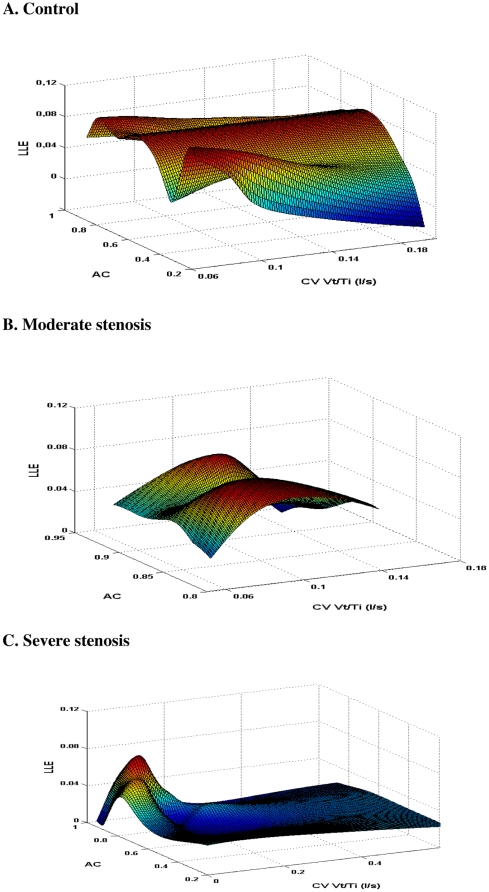
Three dimensional colored parametric surface showing the largest Lyapunov exponent (LLE) (z- axis) as a function of the autocorrelation coefficient (AC) (y-axis) and the coefficient of variation (CV) (x-axis) of inspiratory flow (Vt/Ti) in all subjects of the control group (A), moderate carotid stenosis group (B) and severe carotid stenosis group (C). Color is proportional to the surface height (the value of the LLE). For clarity, the scale of the y-axis (AC) of the middle panel (B) has been narrowed whereas the scale of the x-axis (CV Vt/Ti) of the bottom panel (C) has been enlarged. See text for comments.

Figure 2 shows in a 3Dplot the relationship between the chaotic features of the inspiratory flow, the autocorrelation coefficient and the variability in the 23 controls (top), 12 subjects with moderate stenosis (middle) and 23 with severe stenosis (bottom). In the control group (Figure 2A), there are multiple feedback loops involved in the control of breathing and therefore many subjects exhibit high values of LLE. In the group with moderate stenosis (Figure 2B), breathing occurs with a higher level of linear correlation from breath to breath. Whereas in the group with severe carotid stenosis (Figure 2C), very few subjects display inspiratory chaos and breathing depends predominantly on the linear relations of breath cycle.

### Linear and Nonlinear measures of the ventilatory variables after carotid endarterectomy

**Figure 3:**
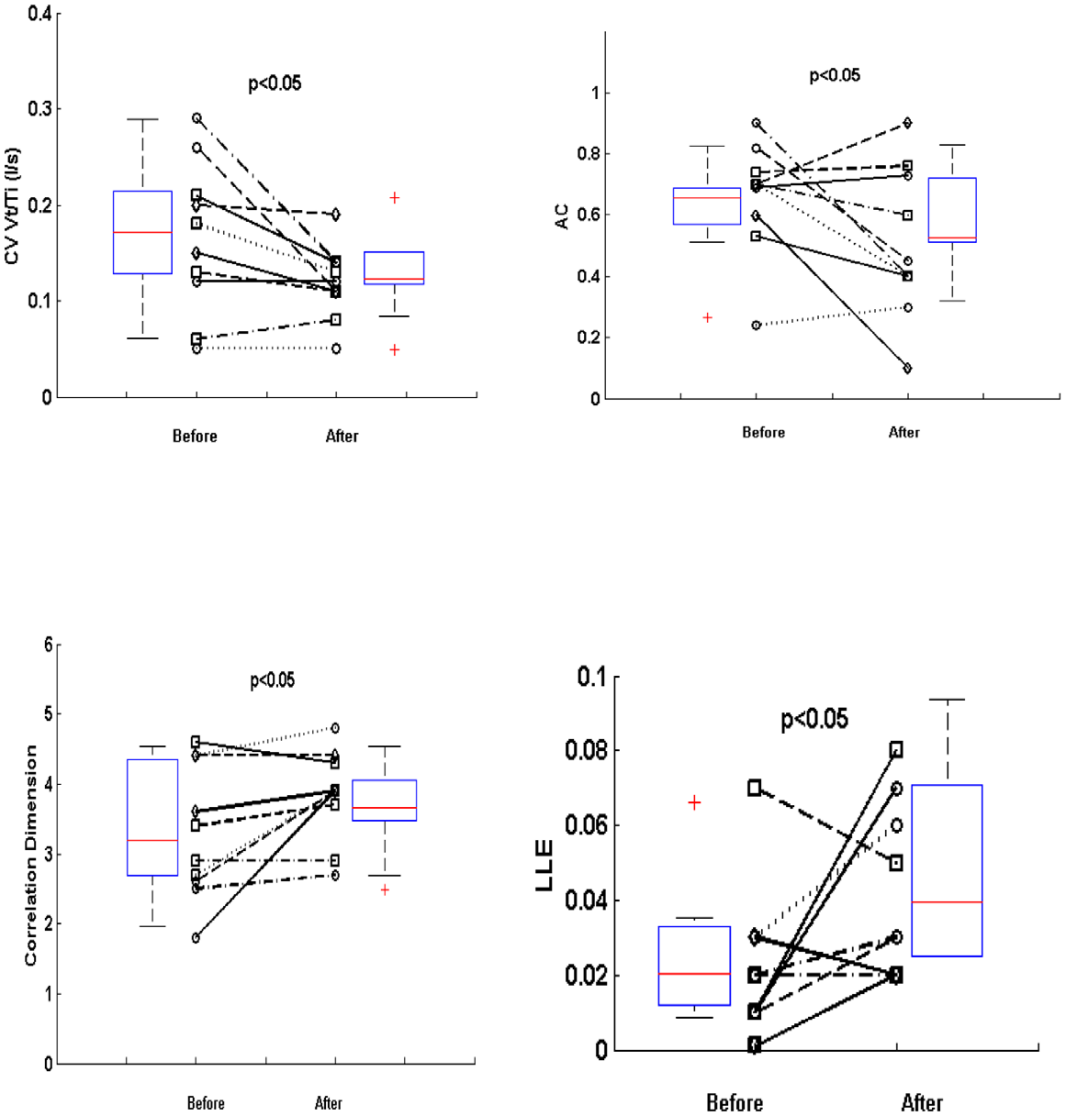
Coefficient of variation, autocorrelation coefficient (one breath lag) (top) and chaotic analysis (correlation dimension and largest Lyapunov exponent) (bottom) of the inspiratory flow before and 72 hours after carotid endarterectomy.

Among the group with severe stenosis, we recorded ventilatory flow in a second series of experiment in 10 subjects, 72 hours after carotid endarterectomy performed under regional anesthesia. As shown in Figure 3, the coefficient of variation and the autocorrelation of the inspiratory flow significantly decreased after removal of the carotid plaque. Chaos was significantly enhanced after endarterectomy, since the correlation dimension and the LLE of the inspiratory flow both increased.

## Discussion

Consistent with our hypotheses, ventilatory chaos is impaired during carotid atherosclerosis. The main determinant of the flow dynamics alteration was the severity of the stenosis although other characteristics of the carotid plaque such as its localization and morphology also significantly decreased ventilatory chaos. Carotid endarterectomy reversed inspiratory flow chaos abnormalities, which reinforced the direct role played by the plaque in these alterations.

### Characterizing ventilatory chaos

Breathing is not a periodic phenomenon but exhibits fluctuations that display information on the breathing control. Nonlinear dynamics in the ventilatory flow output arises as a result of the complex interplay between central processing of the respiratory centers, peripheral afferents [2] and stochastic noise inputs. In this study, we characterized chaos in inspiratory flow time series data with three different methods. We first used the noise titration which exhibits good sensitivity, specificity and robustness in discriminating chaos from random noise. We found a relatively high number of subjects (32%) with carotid stenosis having a null value of the noise titration, which means that the series is not chaotic, or contamined by the noise floor [15]. In addition, the noise limit value was lower in the group with atherosclerosis and similar results were obtained with the two other methods, the correlation dimension that characterizes the aperiodicity of the system and the largest Lyapunov exponent which quantifies the sensitivity to initial conditions. Comparisons were made with surrogate data for the correlation dimension reinforcing the nonlinear feature, a prerequisite for chaos of the inspiratory flow time series.

Breathing pattern abnormalities concern both linear and nonlinear characteristics of the inspiratory flow, as illustrated in Figure 2 which shows in a 3Dplot the relationship between the linear and chaotic features of the inspiratory flow. In the group with moderate and severe stenoses (Figure 2B and C), few patients display high value of LLE. Impairment of the ventilatory control mechanisms may lead to the loss of physiological complexity. Moreover, breathing occurs with a higher level of linear correlation from breath to breath. The autocorrelation analysis represents the relationship between one breath and another at some interval (or lag) away [16], [17]. It determines the relative strength of “short term memory” for each breath component [16]. Our results indicate that the linear predictive part of the breathing pattern increased which means at least a more monotonous pattern of ventilation for these patients. Conversely, controls subjects displayed high values of LLE underlying that the multiple feedback loops involved in the control of breathing increased the chaotic features of the flow.

### Ventilatory chaos is reduced in carotid atherosclerosis

Chaos impairment of inspiratory flow was strongly related to the severity of the carotid stenosis. In multiple regression analyses, the percentage of the carotid stenosis was the best in predicting the chaotic features of the inspiratory flow. However, other characteristics such as localization, surface features and morphology of the plaque also altered ventilatory chaos, independently of the degree of stenosis. The carotid plaques were classified depending on their echogenic features and surface morphology. The homogeneous echogenicity of the plaque is relied on histological characteristic such as collagen-rich, fibrous plaques, while heterogeneous echogenicity is found in lipid-rich or hemorrhagic plaques [18], [19]. Different local vascular consequences depending on the characteristics of the plaque may explain the differences evidenced in the largest Lyapunov exponent and the correlation dimension of the inspiratory flow. The structural changes of the vessel wall composition induce by the plaque (stiffness) directly impairs the baroreceptors [9], [10] and thus modifies the breathing pattern. In addition paracrine factors associated with atherosclerosis may potentiate the effects of structural modulation [20]. Sympathovagal imbalance has also been described during carotid atheroma and this could contribute to ventilatory flow dynamics alterations [10].

Indeed, baroreflex sensitivity has a significant effect in predicting the correlation dimension value and to a lesser extent the LLE of the inspiratory flow in multiple regression models. The baroreceptor reflex controls ventilation through changes in the frequency [12], tidal volume [12], [13] and respiratory variability [14]. Recently, McMullan et al. showed in rats that the variability of the inspiratory and expiratory time, as well as the complexity were altered during aortic baroreceptor activation, at low ventilatory drive [14]. Indeed, baroreceptors afferents project to the nucleus tractus solitarius [21]–[23] and the resulting baroreceptors-mediated effects on central respiratory neural activity and timing consist in changes in respiratory timing and volume.

In patients with severe carotid stenosis, we found that carotid endarterectomy performed under regional anesthesia significantly increased inspiratory flow chaos. This finding pointed out the direct role of the carotid plaque in the ventilatory flow dynamics abnormalities, independently of possible confounding factors such as the age. Plaque removal completely reversed the increased coefficient of variation and autocorrelation coefficient. It also reversed the correlation dimension of the inspiratory flow whereas it partially reversed the LLE (Figure 3).

Vascular alterations, including atherosclerosis and arterial stiffness have been associated with impaired pulmonary function [24], [25]. Reduced peak expiratory flow is linked to the development of carotid atherosclerosis whereas reduced FEV1 and FEV1/FVC are associated with arterial stiffness. The mechanisms underlying these associations are still not entirely understood. Therefore we selected the subjects in our study based on their normal respiratory function to avoid the possible interaction with breathing control [26].

### Study limitations

There is a possible behavioral influence on breathing during wakefulness and the mouthpiece and nose clip we used might have influenced our results [27]. However the experimental conditions were identical for all the groups. In addition, subjects were allowed to comfortably adapt to the conditions for several minutes before the recordings began and the first minute of recording was suppressed from the analysis. Previous report already analyzed the influence of a mouthpiece and noise clip on the ventilatory flow and found it minimal [28]. In addition, we tested for reproducibility of the inspiratory flow measurement in a second series of experiments (Appendix S1) and the Figure S6 illustrates the good reproducibility of our measurements. Another point is that the control group is younger than the group with severe stenosis. It is however unlikely that this could have influence the results since after removing the carotid plaque, the breathing pattern became more similar to that of the normal subjects. We also cautiously excluded patients experiencing stroke after a brain scan because of the previously described breathing pattern abnormalities due to the brain infarction [29]. Finally, we did not evaluate the carotid chemoreceptor function in the subjects. We point out that our study was not design to study all the possible mechanisms involved in the cascade of events during carotid atherosclerosis but to analyze if ventilatory variability and complexity were altered by carotid atheroma. In addition, a previous study already showed that chemoreceptor function was normal in atherosclerotic rabbit although their baroreflexes were altered [30].

### Conclusion

In summary, we showed that ventilatory chaos is impaired in carotid atherosclerosis. These findings depend mainly on the severity of the stenosis, on its localization, plaque surface and morphology features and are independently associated with baroreflex sensitivity reduction. These new findings should help to understand the determinants of ventilatory chaos and breathing control in pathological conditions. It is also possible that besides carotid atheroma, other cardiovascular diseases alter nonlinear dynamics of respiration, and the method may have thus a broad diagnostic value.

## Methods

### Subjects

Consecutive patients, age >50 years, who underwent a carotid ultrasound scan in the Departments of Physiology and Vascular/Thoracic surgery of the Bichat Hospital were included. Other inclusion criteria were: subjects with a history free of pulmonary disease, normal physical examination, chest radiography and pulmonary function tests. Patients with a previous history of transient ischemic attack were also included in the study. Exclusion criteria were respiratory disease, chronic heart failure, stroke and a body mass index above 30 kg/m^2^. Subjects that were taken medications known to interfere with autonomic cardiovascular regulation, β-blockers and calcium inhibitors were also excluded. This research was reviewed and approved by the institutional review board and the ethics committee of the comité de protection des personnes Ile de France 1. All patients gave written informed consent.

### Carotid arteries imaging

High resolution B-mode and Doppler ultrasonography (Vivid 7, General Electrics, USA) of the carotid arteries were performed using a 10-Mhz linear array imaging probe. Trained physicians conducted the ultra-sonographic scanning and interpreted the results. According to vessel narrowing, three groups were identified: Control: no carotid stenosis; moderate stenosis: carotid stenosis below 70% and severe stenosis: carotid stenosis above 70%. This cut-off value was chosen according to ECST criteria concerning significant carotid stenoses [31]. Plaque was categorized according to several surface characteristics: localization (bifurcation/internal carotid, unilateral/bilateral) smoothness (regularity/irregularity) and morphology (homogeneity/heterogeneity) [32]. All the subjects in the group with severe stenosis had a magnetic resonance angiography of the carotid arteries to confirm the degree of the stenosis. A severe stenosis on one side and moderate on the controlateral side was classified as being severe and bilateral.

### Ventilatory and cardiovascular measurements

Subjects were comfortably seated and were asked to keep their eyes open. No particular instructions regarding breathing were given. They wore a nose clip and breathed through a mouthpiece that permitted connection to a pneumotachograph. Noninvasive finger transcutaneous oxygen saturation (Nellcor Pulse oxymeter N200) was checked for every subject before recordings. Recordings were performed during 15–20 minutes at the same time of the day for all subjects. To ensure steady state, the subjects were allowed 5 minutes to adapt to test conditions before the ventilatory flow signal was actually recorded.

Ventilatory flow of the subjects was measured at rest with a low-resistance pneumotachograph linear from 0 to 1000 l/min (MLT 1000L; AD-instruments, Castle Hill, UK; dead space 350ml, flow resistance 0.002cmH20.l^−1^.s). Blood pressure was noninvasively, continuously measured via a plethysmographic finger transducer (AD-Instruments). Ventilatory flow, ECG and blood pressure digitized at 400-Hz sampling rate (PowerLab4/25, AD Insutruments), were synchronously acquired and recorded on a PC computer in the form of data files for subsequent analysis (Chart version 5; AD Instruments). An example of the recorded signals is given in Figure S5. We tested for reproducibility of the inspiratory flow measurements in 10 subjects of group 1 (Appendix S1 and Figure S6).

Among the group with severe stenosis, we recorded ventilatory flow in a second series of experiment in 10 subjects, 72hours after carotid endarterectomy performed under regional anaesthesia.

Lung function tests were performed with a Spyro Analyzer spirometer. The highest value of the forced expiratory volume in the first second (FEV_1_) was given and the ratio of FEV1 to FVC (forced vital capacity) (% FEV1/FVC).

#### Analysis of ventilation in the time domain and autocorrelation analysis

The first minute of recording was suppressed from the analysis. Each following ventilatory variable was computed on a breath-by-breath basis from the ventilatory flow signal: inspiratory time (Ti), expiratory time (Te), total cycle time (Ttot), tidal volume (Vt), inspiratory flow (Vt/Ti), inspiratory duty cycle (Ti/Ttot). The variability of these indices was evaluated through their coefficients of variation (the ratio of the standard variation to the mean). Autocorrelated fraction of the inspiratory flow was computed at a lag of one breath (Matlab 7.7, Mathworks USA). Autocorrelated fraction of breath components has been validated as a mean to assess the correlated linear part of the flow [17].

#### Nonlinear analyses

The noise titration technique [15] was used on the inspiratory flow time series described above. It is a highly sensitive, specific and robust detection of chaos in short noisy data. We already used the method on experimental time series to evidence the chaotic nature of human ventilation [2], [6]. First, the method involved the simulation of time series with linear and nonlinear polynomial autoregressive model (Volterra-Wiener series) [33]. The best linear and nonlinear models are chosen according to the minimal information theoretic criterion. The null hypothesis, a stochastic time series with linear dynamics, was rejected if the best nonlinear model provided a significant better fit to the data than the best linear model using parametrics (F-test) statistics at the 1% significance level. Once nonlinear determinism was indicated, white noise of increasing standard deviation was added to the data until nonlinearity could no longer be detected, i.e. the nonlinearity was ‘neutralized’. The noise limit (NL) was calculated as the percent of signal power added as noise to ‘titrate’ the data to the point of neutrality. Typically, an average NL value was obtained by repeating the titration procedure 5–10 times. Under this scheme, chaos is indicated by NL >0, and the value of NL provides a relative measure of chaos intensity. Conversely, if NL  = 0, then it may be inferred that the series either is not chaotic or the chaotic component is already neutralized by the background noise (noise floor) in the data. We then estimated the largest Lyapunov exponent and the correlation dimension of the time series having a positive noise limit value.

Complex dynamical systems are sensitive to initial conditions, and exhibit an exponential divergence in the phase space. This can be quantified through the study of the Lyapunov exponents spectrum and the calculation of the largest Lyapunov exponent (λ_L_: LLE). Consider two points on two nearby trajectories in the phase space, and assume the distance between them to be d(0). After time t, if the distance between the two trajectories becomes d(t), then the average divergence (separation after time t) can be written as : 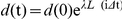
where λ*_L_* is the LLE of the system. In the present study, we used the algorithm proposed by Rosenstein et al that has been shown to be particularly useful for small data series [34].

The correlation dimension (D_corr_) is a fractal dimension reflecting the irregularity of the attractor of the system. D_corr_ characterizes the “aperiodicity” of the system in the phase space. It is estimated by examining the scaling properties of the correlation sum. From a time series (x1, x2,…x_N_), where N is the total number of points, the m dimensional vector in the phase space can be constructed by delay embedding:
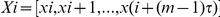
where, τ is the fixed time lag and m is the embedding dimension. Then the reconstructed trajectory of the actual dynamics can be written as *X*  =  (X_1_; X_2_; X_3_; …X*_M_*), where M = N–(m-1)_τ_.

The correlation dimension can be calculated from the correlation integral of the time series. The correlation integral can be computed as follows [35], [36]:
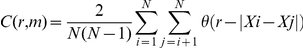
where, r is scale length, and θ is the Heavyside step function. Scaling of the function *C(r,m)* can be written as: 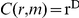.

The correlation dimension (D_corr_) can be defined by
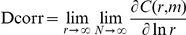
and for practical purpose, D_corr_ can be obtained from the slope of ln *C(r)* vs ln *r* plot.

Time lag was first estimated by a drop of the autocorrelation to (1-1/e) [34], [36]. If an appropriate lag could not be obtained, then the mutual information method allows the calculation of the time delay [36]. The optimal dimension was obtained after calculating the percentage of false nearest neighbors between points in phase space. A minimal number of false nearest neighbors was required [37]. The embedding dimension that adequately represents the system is the dimension that eliminates most of the false nearest neighbors allowing an adequate phase-space reconstruction of the underlying dynamics [37]. An appropriate time lag and embedding dimension were estimated for each experimental time series.

In order to test the nonlinearity that governs the dynamics, we have applied surrogate test [38]. First the Fourier transform of the original time series is computed. Then the phase is replaced by random numbers and finally the inverse Fourier transform is applied. Power spectrum is thus preserved although the nonlinear structures are destroyed [38]. Correlation dimension have been estimated for both the original data and five surrogates that match each original signal. A global test was carried out by a Wilcoxon signed-rank test comparing the correlation dimension values computed on the original data paired with the corresponding average correlation dimension values form the matching surrogate. Significant Wilcoxon rank test between the original and surrogates implies the nonlinear dynamics of the original data.

#### Autonomic nervous system activity analyzed through baroreflex sensitivity

From the continuous ECG and BP recordings, RR intervals and systolic blood pressure time series were computed. Stationary segments of 15-minutes recordings free of artifact and ectopic beats were selected. Equidistant resampling, linear interpolation and power spectral analysis were performed using the Welch's algorithm applied to a 256 Hanning window with 50% overlap. The overall baroreflex gain was computed as the square roots of the ratio of the power spectral density of the RR and systolic blood pressure spectra (α index) [39]. This index accurately reflects baroreflex sensitivity when the coherence value between the two spectra is above 0.5.

### Statistical analyses

Matlab R2010a was used for statistical and signal processing analyses (Mathworks, USA). Comparisons between clinical data among the three groups were made using univariate analysis and the χ^2^ test. The normality of the distributions of the discrete respiratory variables was ascertained using the Kolmogorov-Smirnov test. The occurrence of a positive noise limit in the time series was compared using the χ^2^ test. One way and N-way analysis of variance were used to study statistical differences of the linear and nonlinear measures of the inspiratory flow among the three groups of carotid stenosis. N-way analyses of variance were used to analyze the main significant effects and interactions among the following variables: percentage of carotid stenosis, characterization of the plaque (regularity, homogeneity) and localization (bifurcation or internal carotid, unilateral or bilateral) of the plaque. Multiple comparisons were performed using the “Tukey-Kramer” test. Partial correlation and multiple regression analyses were performed to study the strength of the relation between ventilatory variables and the degree of carotid stenosis and the baroreflex sensitivity controlling for age.

## Supporting Information

Figure S1 Coefficient of variation (CV) of the inspiratory time (Ti), total cycle time (Ttot), expiratory time (Te), tidal volume (Vt) and duty cycle (Ti/Ttot) in control subjects in case of an unilateral and bilateral stenoses. The boxes encompass the interquartile range with indication of the median, the whiskers delimit the 95^th^ percentile of the data distribution (univariate analysis).
(TIF)

Figure S2 Coefficient of variation (CV) of the inspiratory time (Ti), total cycle time (Ttot), expiratory time (Te), tidal volume (Vt) and duty cycle (Ti/Ttot) and inspiratory flow (Vt/Ti) in control subjects in case of an internal carotid and bifurcation stenoses. The boxes encompass the interquartile range with indication of the median, the whiskers delimit the 95^th^ percentile of the data distribution (univariate analysis).
(TIF)

Figure S3 Coefficient of variation (CV) of the inspiratory time (Ti), total cycle time (Ttot), expiratory time (Te), tidal volume (Vt) and duty cycle (Ti/Ttot) in control subjects in case of an regular/irregular (left panel) and homogeneous/heterogeneous (right panel) carotid stenoses. The boxes encompass the interquartile range with indication of the median, the whiskers delimit the 95^th^ percentile of the data distribution (univariate analysis).
(TIF)

Figure S4 Chaos characterization of the inspiratory flow with the largest Lyapunov exponent (LLE) (left panel) and correlation dimension (right panel) according to unilateral/bilateral carotid stenoses (top), internal carotid/bifurcation stenoses (middle), regular/irregular stenoses (middle) and homogeneous/heterogeneous stenoses (bottom). The boxes encompass the interquartile range with indication of the median, the whiskers delimit the 95^th^ percentile of the data distribution (univariate analysis).
(TIF)

Figure S5 Signals acquisition for thirty seconds of the ventilatory flow (top), blood pressure (middle) and ECG (bottom) in one subject. X-axis is in sec.
(TIF)

Figure S6 Reproducibility of the inspiratory flow measurements in ten subjects of group1. The second measurement was made 72 hours after, with the same experimental conditions. Mean value of inspiratory flow (Vt/Ti) at the top, coefficient of variation (CV) and autocorrelation coefficient (AC) of the inspiratory flow in the middle, and noise limit value at the bottom. The boxes encompass the interquartile range with indication of the median, the whiskers delimit the 95^th^ percentile of the data distribution.
(TIF)

Appendix S1 Reproducibility of the inspiratory flow measurements.
(DOC)
