# Supplementary figures and images for: Ventilatory Chaos Is Impaired in Carotid Atherosclerosis
Source: PLoS One. 2011 Jan 28;6(1):e16297. doi: 10.1371/journal.pone.0016297 (PMC3030574; doi:10.1371/journal.pone.0016297)

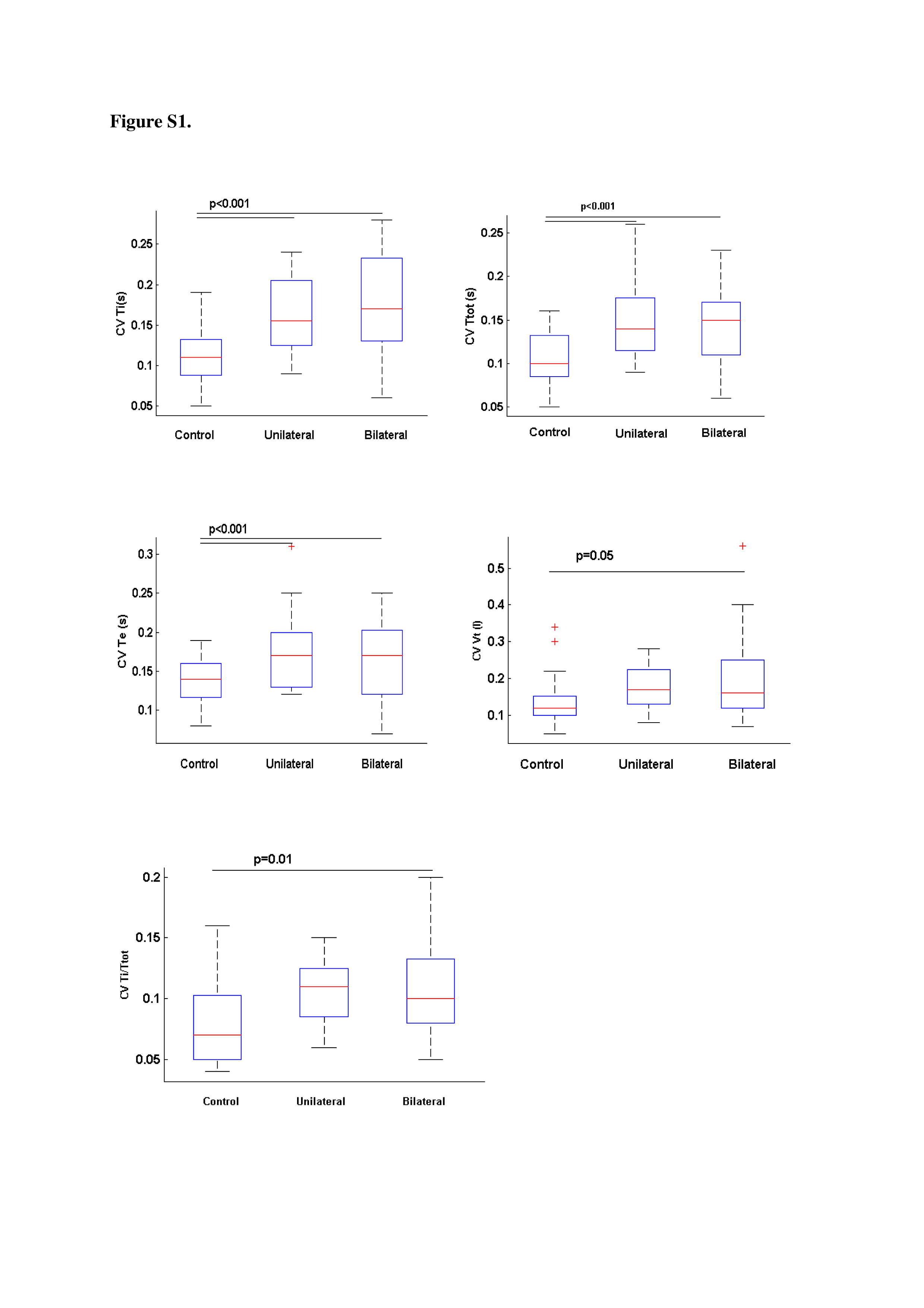

Supplement: Figure S1 — Coefficient of variation (CV) of the inspiratory time (Ti), total cycle time (Ttot), expiratory time (Te), tidal volume (Vt) and duty cycle (Ti/Ttot) in control subjects in case of an unilateral and bilateral stenoses. The boxes encompass the interquartile range with indication of the median, the whiskers delimit the 95th percentile of the data distribution (univariate analysis). (TIF) [file pone.0016297.s001.tif]

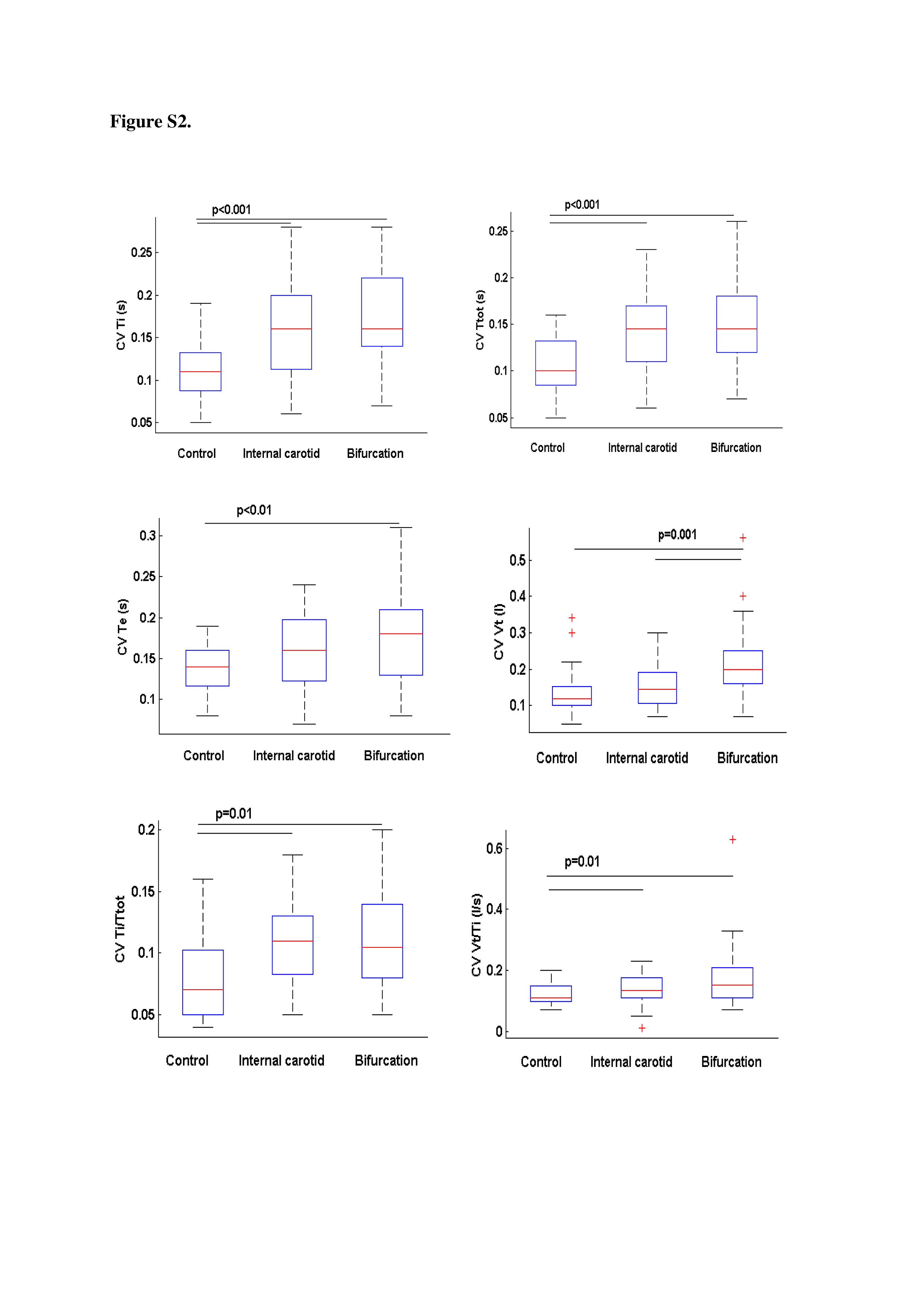

Supplement: Figure S2 — Coefficient of variation (CV) of the inspiratory time (Ti), total cycle time (Ttot), expiratory time (Te), tidal volume (Vt) and duty cycle (Ti/Ttot) and inspiratory flow (Vt/Ti) in control subjects in case of an internal carotid and bifurcation stenoses. The boxes encompass the interquartile range with indication of the median, the whiskers delimit the 95th percentile of the data distribution (univariate analysis). (TIF) [file pone.0016297.s002.tif]

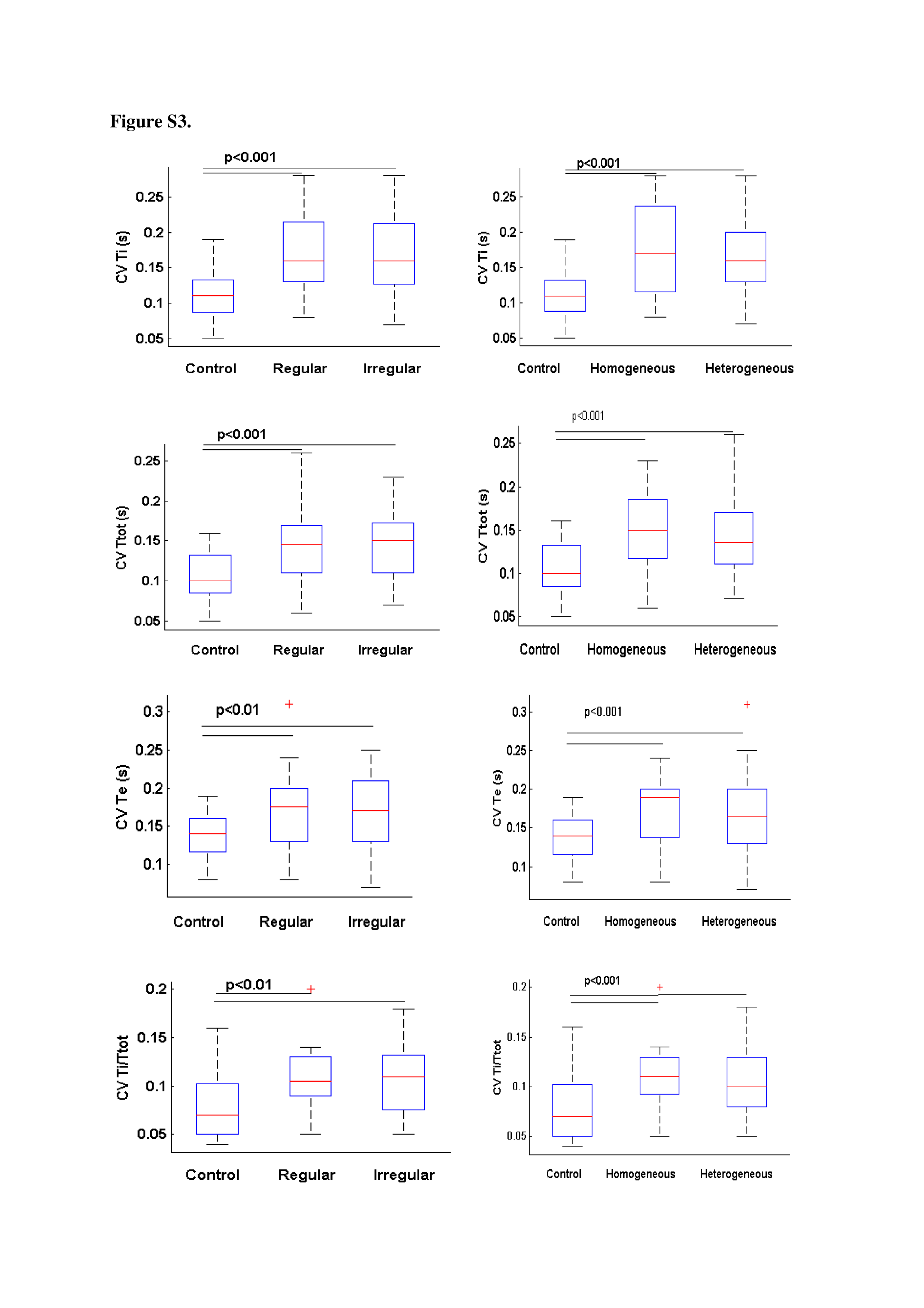

Supplement: Figure S3 — Coefficient of variation (CV) of the inspiratory time (Ti), total cycle time (Ttot), expiratory time (Te), tidal volume (Vt) and duty cycle (Ti/Ttot) in control subjects in case of an regular/irregular (left panel) and homogeneous/heterogeneous (right panel) carotid stenoses. The boxes encompass the interquartile range with indication of the median, the whiskers delimit the 95th percentile of the data distribution (univariate analysis). (TIF) [file pone.0016297.s003.tif]

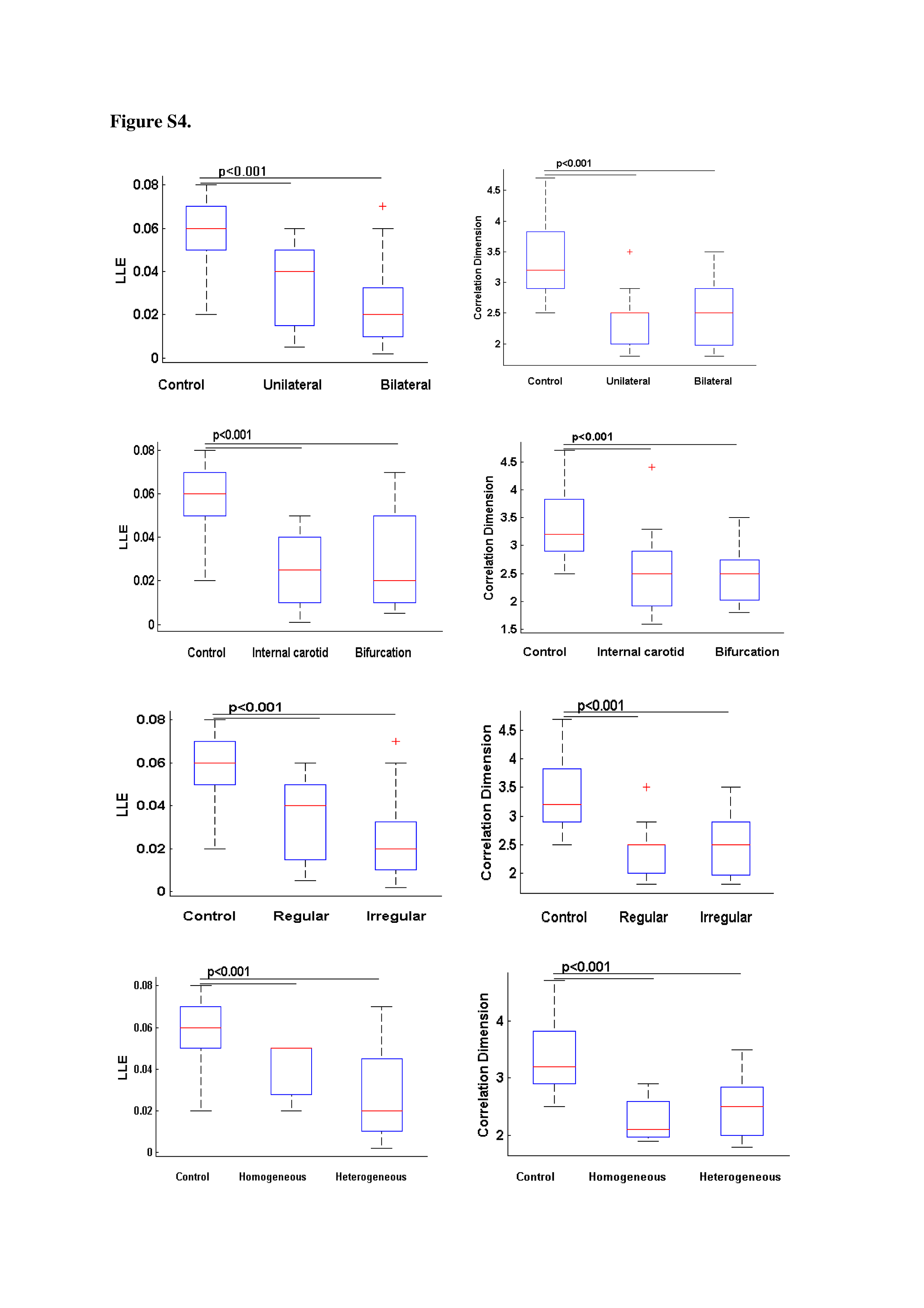

Supplement: Figure S4 — Chaos characterization of the inspiratory flow with the largest Lyapunov exponent (LLE) (left panel) and correlation dimension (right panel) according to unilateral/bilateral carotid stenoses (top), internal carotid/bifurcation stenoses (middle), regular/irregular stenoses (middle) and homogeneous/heterogeneous stenoses (bottom). The boxes encompass the interquartile range with indication of the median, the whiskers delimit the 95th percentile of the data distribution (univariate analysis). (TIF) [file pone.0016297.s004.tif]

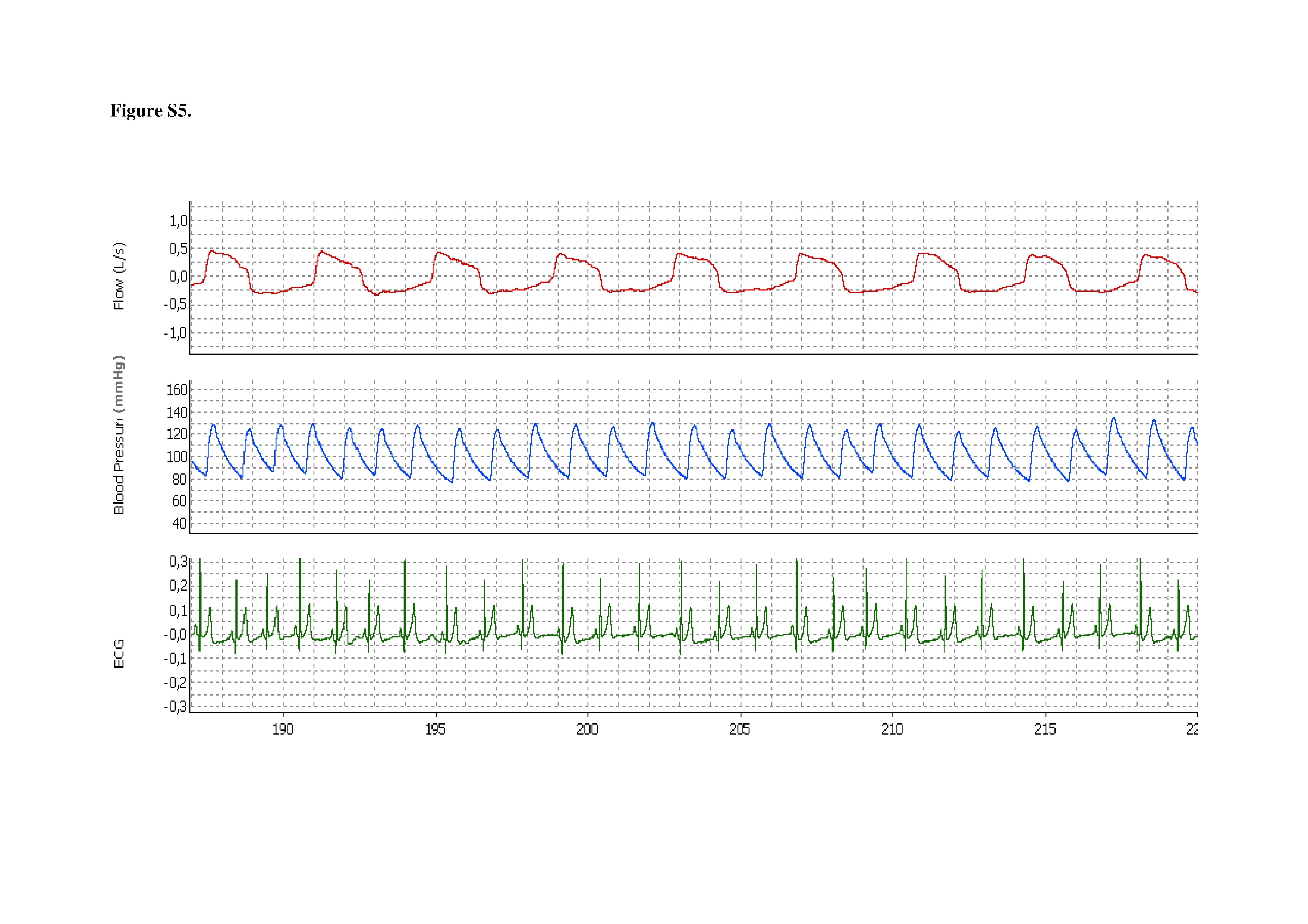

Supplement: Figure S5 — Signals acquisition for thirty seconds of the ventilatory flow (top), blood pressure (middle) and ECG (bottom) in one subject. X-axis is in sec. (TIF) [file pone.0016297.s005.tif]

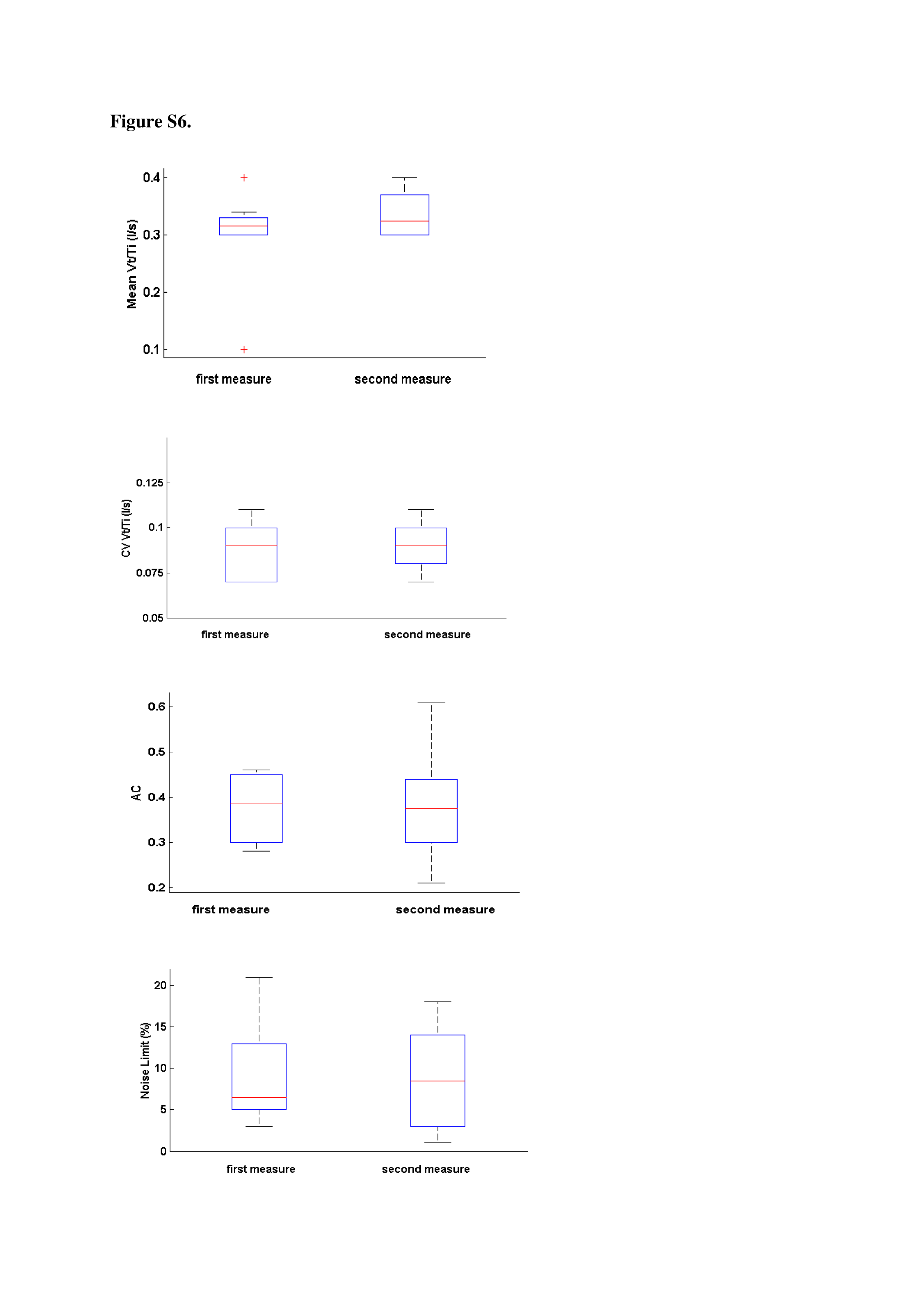

Supplement: Figure S6 — Reproducibility of the inspiratory flow measurements in ten subjects of group1. The second measurement was made 72 hours after, with the same experimental conditions. Mean value of inspiratory flow (Vt/Ti) at the top, coefficient of variation (CV) and autocorrelation coefficient (AC) of the inspiratory flow in the middle, and noise limit value at the bottom. The boxes encompass the interquartile range with indication of the median, the whiskers delimit the 95th percentile of the data distribution. (TIF) [file pone.0016297.s006.tif]
