# Supplementary material for: Ventilatory Chaos Is Impaired in Carotid Atherosclerosis
Source: PLoS One. 2011 Jan 28;6(1):e16297. doi: 10.1371/journal.pone.0016297 (PMC3030574; doi:10.1371/journal.pone.0016297)
Supplement: Appendix S1 — Reproducibility of the inspiratory flow measurements. (DOC) [file pone.0016297.s007.doc]

**Appendix 1.**

We test for ventilatory flow measurement reproducibility in two series of experiment in ten subjects of group 1. Second measurements were performed 72 hours later in the same conditions. Subjects were comfortably seated and were asked to keep their eyes open. No particular instructions regarding breathing were given. They wore a nose clip and breathed through a mouthpiece that permitted connection to a pneumotachograph. Noninvasive finger transcutaneous oxygen saturation (Nellcor Pulse oxymeter N200) was checked for every subject before recordings. Recordings were performed during 15-20 minutes at the same time of the day for all subjects (for the first and second measurements). To ensure steady state, the subjects were allowed 5 minutes to adapt to test conditions before the ventilatory flow signal was actually recorded. Ventilatory flow of the subjects was measured at rest with a low-resistance pneumotachograph linear from 0 to 1000 l/min (MLT 1000L; AD-instruments, Castle Hill, UK; dead space 350ml, flow resistance 0.002cmH20.l^-1^.s). Signal was digitized at 400-Hz sampling rate (PowerLab4/25, AD Insutruments) and was recorded on a PC computer in the form of data files for subsequent analysis (Chart version 5; AD Instruments). Mean values and the coefficients of variation of inspiratory time (Ti), expiratory time (Te), total cycle time (Ttot), tidal volume (Vt), inspiratory flow (Vt/Ti), inspiratory duty cycle (Ti/Ttot) were estimated, as autocorrelated fraction of breath components and chaos detection of inspiratory flow (noise titration). As shown in Figure S6, good reproducibility was achieved between the two ventilatory measurements.
